# *Ex Vivo* Expanded Human Non-Cytotoxic CD8^+^CD45RC^low/−^ Tregs Efficiently Delay Skin Graft Rejection and GVHD in Humanized Mice

**DOI:** 10.3389/fimmu.2017.02014

**Published:** 2018-01-31

**Authors:** Séverine Bézie, Dimitri Meistermann, Laetitia Boucault, Stéphanie Kilens, Johanna Zoppi, Elodie Autrusseau, Audrey Donnart, Véronique Nerrière-Daguin, Frédérique Bellier-Waast, Eric Charpentier, Franck Duteille, Laurent David, Ignacio Anegon, Carole Guillonneau

**Affiliations:** ^1^Centre de Recherche en Transplantation et Immunologie UMR1064, INSERM, Université de Nantes, Nantes, France; ^2^Institut de Transplantation Urologie Néphrologie (ITUN), CHU Nantes, Nantes, France; ^3^LabEx IGO “Immunotherapy, Graft, Oncology”, Nantes, France; ^4^Laboratoire des Sciences du Numérique de Nantes (LS2N) UMR6004, Université de Nantes, Nantes, France; ^5^INSERM UMR1087, CNRS UMR6291, Université de Nantes, l’institut du thorax, Nantes, France; ^6^Chirurgie Plastique Reconstructrice et Esthétique, CHU Nantes, Nantes, France; ^7^INSERM UMS 016, SFR Francois Bonamy, iPSC core facility, CNRS UMS 3556, Université de Nantes, CHU de Nantes, Nantes, France

**Keywords:** transplantation, tolerance, Treg, cell therapy, graft, GVHD, NSG mice

## Abstract

Both CD4^+^ and CD8^+^ Tregs play a critical role in the control of immune responses and immune tolerance; however, our understanding of CD8^+^ Tregs is limited while they are particularly promising for therapeutic application. We report here existence of highly suppressive human CD8^+^CD45RC^low/−^ Tregs expressing Foxp3 and producing IFNγ, IL-10, IL-34, and TGFβ to mediate their suppressive activity. We demonstrate that total CD8^+^CD45RC^low/−^ Tregs can be efficiently expanded in the presence of anti-CD3/28 mAbs, high-dose IL-2 and IL-15 and that such expanded Tregs efficiently delay GVHD and human skin transplantation rejection in immune humanized mice. Robustly expanded CD8^+^ Tregs displayed a specific gene signature, upregulated cytokines and expansion in the presence of rapamycin greatly improved proliferation and suppression. We show that CD8^+^CD45RC^low/−^ Tregs are equivalent to canonical CD4^+^CD25^high^CD127^low/−^ Tregs for suppression of allogeneic immune responses *in vitro*. Altogether, our results open new perspectives to tolerogenic strategies in human solid organ transplantation and GVHD.

## Introduction

Immunosuppressive regimens have significantly improved long-term graft survival in the last decades but they still cannot prevent the allograft from chronic graft dysfunction and they remain a significant obstacle for the welfare of transplanted patients, thus, in the last years, improvement of allograft survival has stagnated (1). The identification in human of regulatory cell populations actively controlling immune responses in transplantation with high suppressive capacity and specificity toward donor antigens has generated revolutionizing therapeutic strategies in a number of diseases with a Treg/effector T cells (Teff) deregulation. The establishment of cellular therapy with regulatory cells has recently emerged as a promising future therapy in autoimmunity as well as bone marrow and solid organ transplantation (2–4). Phase I studies in GVHD and solid organ transplantation have started with regulatory cells from different types (different CD4^+^ Tregs, macrophages, and DCs) without apparent toxicity (5–7), but to date, there are no clinical trials using CD8^+^ Tregs despite abundant literature in animals models (8–10). One limitation for translation of CD8^+^ Tregs in humans might be the lack of recognized marker such as Foxp3, a critical gene in the function of canonical CD4^+^CD25^high^CD127^low/−^ Tregs (11, 12). However, the role and expression of Foxp3 has not been clearly defined for CD8^+^ Tregs, and its function has not been clearly demonstrated (9, 13–20). Others have shown in mice that adoptive transfer of antigen-specific CD8^+^ Tregs were potent suppressors of fully MHC mismatch skin allograft and islet allograft (21, 22). CD122^+^PD-1^+^CD8^+^ Tregs efficiently inhibited skin allograft rejection in mice upon adoptive transfer and were more efficient at inhibiting islets allograft rejection than CD4^+^CD25^+^ Tregs (23, 24). Our own studies have shown in a rat model of CD40-CD40L blockade-induced allograft tolerance the critical role of a CD8^+^ Tregs population expressing low/no level of CD45RC (8, 25). Furthermore, treatment with anti-CD45RC depleted CD45RC^high^ cells, preserved CD45RC^low/−^ CD8^+^ and CD4^+^ Tregs and resulted in inhibition of solid organ rejection and of human GVHD in immune humanized mice (25). We highlighted the biological role of IFNγ, IL-34, and Fibroleukin-2 (FGL-2) in the suppression exerted by CD8^+^CD45RC^low/−^ Tregs (8, 17, 26–28).

In the present article, we aim to further characterize human CD8^+^ Tregs, to assess their potential for cell therapy in solid organ and bone marrow transplantation. We demonstrate here by extensive flow cytometry phenotyping, 3′digital gene expression (3′DGE) RNA-sequencing (DGE-RNAseq) and *in vitro* suppressive assays, the high suppressive capacity of a subset of CD8^+^CD45RC^low/−^ Tregs expressing Foxp3, GITR, IL-10, IL-34, TGFβ, and IFNγ at low levels in contrast to CD8^+^CD45RC^high^ T cells. We demonstrate that CD8^+^CD45RC^low/−^ Tregs suppress CD4^+^ effector T cell immune responses equivalently to canonical CD4^+^CD25^high^CD127^low/−^ Tregs. We demonstrate that total CD8^+^CD45RC^low/−^ Tregs can be efficiently expanded in the presence of anti-CD3/28, high-dose IL-2 and IL-15. We demonstrate that expanded CD8^+^CD45RC^low/−^ Tregs expressed Foxp3^+^ and high amount of cytokines and displayed high suppressive potential in immune humanized mice for both GVHD and human skin transplantation models. We show that expansion in the presence of rapamycin (Rapa) increased both the expansion yield and suppressive capacity of CD8^+^CD45RC^low/−^ Tregs. Analysis of CD8^+^CD45RC^low/−^ Tregs following expansion showed a selective gene signature both at transcriptomic and proteomic levels. Altogether our study highlights the unappreciated potential of CD8^+^ Tregs to control rejection in solid organ transplantation and GVHD.

## Materials and Methods

### Healthy Volunteers Blood Collection and PBMCs Separation

Blood was collected at the Etablissement Français du Sang (Nantes, France) from healthy individuals (Figure S1A,B in Supplementary Material). Written informed consent was provided according to institutional guidelines. PBMCs were isolated by Ficoll–Paque density-gradient centrifugation (Eurobio, Courtaboeuf, France). Remaining red cells and platelets were eliminated with a hypotonic solution and centrifugation. When indicated, PBMCs were frozen in DMSO:FCS 1:9.

### Cell Isolation

Peripheral blood lymphocytes were obtained from PBMCs by elutriation (DTC Plateforme, Nantes) and T cells were purified by magnetic depletion (Dynabeads, Invitrogen) of CD19^+^, CD14^+^ and CD16^+^ cells before sorting of CD3^+^CD4^+^CD25^−^ cells as responder cells, CD3^+^CD4^−^CD45RC^low/−^ as CD8^+^Tregs and CD3^+^CD4^+^CD25^high^CD127^low/−^ cells as CD4^+^Tregs cells with FACS ARIA I (BD Biosciences, Mountain View, CA, USA). Tregs were stimulated overnight with plastic coated with anti-CD3 (OKT3 clone, 1 µg/ml) and soluble anti-CD28 (CD28.2 clone, 1 µg/ml) mAbs in the presence of 250 U/ml IL-2 before plating, or before sorting on IFNγ and/or IL-10 expression using secretion assay detection kits (Miltenyi). APCs were obtained by magnetic depletion of CD3^+^ cells and 35 Gy irradiation. Plasmacytoid dendritic cells (pDCs) and conventional dendritic cells (cDCs) were obtained by CD3, CD14, CD16, and CD19 positive cells depletion and Nrp1^+^ (also known as CD304 or BDCA-4) or CD1c^+^ cell sorting, respectively (29, 30). Purity following sorting was always >97%.

### Monoclonal Antibodies and Flow Cytometry

Antibodies used are listed in Table 1. For stimulation, PBMCs were incubated with PMA (50 ng/ml) and ionomycine (1 µg/ml) for 7 h in the presence of Brefeldine A (10 µg/ml). Fc Receptors were blocked (BD Biosciences) and cells were permeabilized with Fix/Perm kit (Ebiosciences).

**Table 1:** List of antibodies used for phenotypic characterization and sorting by flow cytometry.

Marker	Clone	Provider
2B4	2–69	BD Biosciences
BTLA	REA224	Miltenyi
	J168-540	BD Biosciences
CD103	Ber-ACT8	BD Biosciences
CD122	Mik-β2	BD Biosciences
CD124	hIL4R-M57	BD Biosciences
CD127	HIL-7R-M21	BD Biosciences
CD137	4B4-1	BD Biosciences
CD14	M5E2	BD Biosciences
CD154	TRAP1	BD Biosciences
CD160	BY55	BD Biosciences
CD19	HIB19	BD Biosciences
CD197	3D12	BD Biosciences
CD1c	AD5-8E7	Miltenyi
CD25	BC96	ebiosciences
	M-A251	BD Biosciences
CD27	L128	BD Biosciences
	M-T271	BD Biosciences
CD28	CD28.2	BD Biosciences
CD297 PD1	EH12.1	BD Biosciences
CD3	UCHT1	BD Biosciences
	SK7	BD Biosciences
CD38	HIT2	BD Biosciences
CD39	TU66	BD Biosciences
CD4	RPA-T4	BD Biosciences
	259D/C7	BD Biosciences
CD45RA	HI100	BD Biosciences
CD45RC	MT2	Mast Diagnostic
CD45RO	UCHL1	BD Biosciences
CD56	B159	BD Biosciences
CD62L	DREG-56	BD Biosciences
CD69	FN50	BD Biosciences
CD71	M-A712	BD Biosciences
CD8	SK1	BD Biosciences
	RPA-T8	BD Biosciences
CTLA4	BNI3	BD Biosciences
Foxp3	236A/E7	ebiosciences
	259D/C7	BD Biosciences
GITR	DT5D3	Miltenyi
GP49	ZM4.1	ebiosciences
GZM B D48	GB1	BD Biosciences
HLA DR	G46-6	BD Biosciences
ICOS	ISA-3	ebiosciences
IFNg	B27	BD Biosciences
IL10	JES3-19F1	BD Biosciences
IL17A	eBio64DEC17	ebiosciences
IL2	5344.111	BD Biosciences
IL34	#578416	Biotechne
LAG3	T47-530	BD Biosciences
Nrp1	U21-1283	BD Biosciences
PD1	EH12.1	BD Biosciences
Perforin	B-D48	Diaclone
Tbet	O4-46	BD Biosciences
TGFb1	TW4-9E7	BD Biosciences
TIM3	7D3	BD Biosciences

Fluorescence was measured with a LSR II or a Canto II cytometer (BD Biosciences, Mountain View, CA, USA) and analyzed with FLOWJO software (Tree Star, Inc., Ashland, OR, USA).

### CpG Methylation of Foxp3

Pyrosequencing of bisulfite-modified genomic DNA was used to determine CpG methylation. Methylation analysis was conducted by EpigenDx for Foxp3 (Human FOXP3—ADS3576—ADS783) (http://epigendx.com/d/).

### Mixed Lymphocyte Reaction

CD8^+^CD45RC^low/−^ Tregs suppressive activity was assessed on syngeneic responder CD4^+^CD25^−^ T cells stimulated with allogeneic APCs, cDCs, or pDCs, at 1:1 ratio. CD8^+^CD45RC^low/−^ Tregs were stimulated overnight with coated anti-CD3 and soluble anti-CD28 MAbs (1 µg/ml each) in medium supplemented with 250U/ml IL-2 (Proleukin^®^, Novartis) when indicated. 1,000 U/ml IL-2 (Proleukin^®^, Novartis) or 50 µg/ml blocking mAbs or isotypic control mAbs were added at day 0 (Table 1). Transwell membrane (0.4 µM pores) (ThermoFisher Scientific) was used. Proliferation of CFSE-labeled (ThermoFisher Scientific) CD4^+^ responder cells was analyzed by flow cytometry after 5 days of coculture in complete RPMI1640 medium supplemented with 5% AB serum, by gating on CD3^+^CD4^+^ living cells (DAPI negative) and excluding CPD-V450 (ThermoFisher Scientific) labeled CD4^+^Tregs.

### Cytotoxicity Assay

CD8^+^CD45RC^low/−^ Tregs were used as effector cells for lysis of syngeneic CD4^+^CD25^−^ T cells or allogeneic APCs. After 15 h coculture in 5% AB serum culture medium, specific lysis was assessed by flow cytometry by analyzing Annexin V (BD Biosciences) and DAPI on CD3^+^CD4^+^ or CD3^−^ target cells. Percent of specific lysis was calculated as (% lysis − % spontaneous lysis)/(% maximum lysis − % spontaneous lysis) × 100.

### 3′DGE RNA-Sequencing

RNeasy-Mini Kits (Qiagen) were used to isolate total RNA that was then processed for RNA sequencing. Protocol of 3′DGE RNA-sequencing was performed as previously described (25). ENA Study accession number PRJEB20793.

### Expansion of CD8^+^CD45RC^low/−^ Tregs

CD8^+^CD45RC^low/−^ Tregs were seeded at 3 × 10^5^/ml in complete RPMI1640 medium 10% AB serum, IL-2 (1,000 U/ml) and IL-15 (10 ng/ml), coated anti-CD3 mAb (1 µg/ml), soluble anti-CD28 mAb (1 µg/ml), and/or allogeneic APCs at 1:4 Tregs:APCs ratio. At day 7, expanded cells were diluted at 1.5 × 10^5^/ml and stimulated again. IL-2 and IL-15 cytokines were freshly added at days 0, 7, 10, and 12. Cyclosporine A (CsA, 45 ng/ml), Rapa (45 ng/ml), methylprednisolone (MPr, 500 pg/ml), tacrolimus (2 ng/ml), or mycophenolate mofetil (MPA, 1 µg/ml) were used (31–35). Suppressive activity was tested on CD8^+^CD45RC^low/−^ Tregs expanded more than 10-fold in 7 days. For long-term expansion, Tregs were stimulated again with coated anti-CD3 (1 µg/ml), soluble anti-CD28 MAbs (1 µg/ml) at days 14 and 21 and IL-2 and IL-15 cytokines were added every 2 days from days 7 to 28.

### Humanized Mouse Models

The 8–12-week-old NOD/SCID/IL2Rγ^−/−^ (NSG) mice were bred in our own animal facilities in SPF conditions (accreditation number C44-278) and this study was carried out according to permit number APAFIS 3168 from ministry of research.

For xenogeneic GVHD experiments, 1.5 × 10^7^ fresh PBMCs were intravenously injected with or without polyclonally expanded syngeneic CD8^+^CD45RC^low/−^ Tregs in 1.5 Gy-irradiated NSG mice. Human PBMCs engraftment was monitored in blood and GVHD development was evaluated by weight loss.

For skin transplantation, human skins were obtained from healthy donors from abdominoplasty surgery and transplantation was performed as previously described (36). 5 × 10^6^ PBMCs allogeneic to the graft were injected intravenously with or without syngeneic 14 days expanded CD8^+^CD45RC^low/−^ Tregs. A graft rejection score from 0 to 3 was based on dryness (score 1), rigidity (score 2) and scab (score 3) by macroscopic observation.

### Statistical Analysis

#### Wilcoxon signed-rank test

Wilcoxon matched-pairs signed rank test was used to compare the phenotype of CD8^+^ T cells subpopulations applying a non-parametric *t*-test (*n* ≤ 50) with matched observations (values among CD45RC^high vs low^ for each individual).

#### Mann–Whitney

Mann–Whitney was used to compare Tregs proportion in healthy volunteer populations, and to compare Tregs yield and function after different expansion protocols. Mann–Whitney was used to compare two unpaired sets of values applying a non parametric test (*n* < 50).

#### Two-Way Row-Matched (RM)

Two-way RM ANOVA was used to compare function of cell populations in a range of cell ratio (two factors were type of suppressor cell and cell ratios) and to analyze graft rejection score and mice weight loss over time (two factors were the time and treatment). Bonferroni post test was used to compare suppressive activity for each ratio.

#### Log Rank (Mantel Cox) Test

Log rank (Mantel Cox) test was used to analyze *in vivo* survival.

## Results

### Non-Cytotoxic Human CD8^+^CD45RC^low/−^ T Cells, but Not CD8^+^CD45RC^high^ T Cells, Efficiently Inhibit Allogeneic Immune Responses in a Contact-Dependent Manner

**Figure 1:**
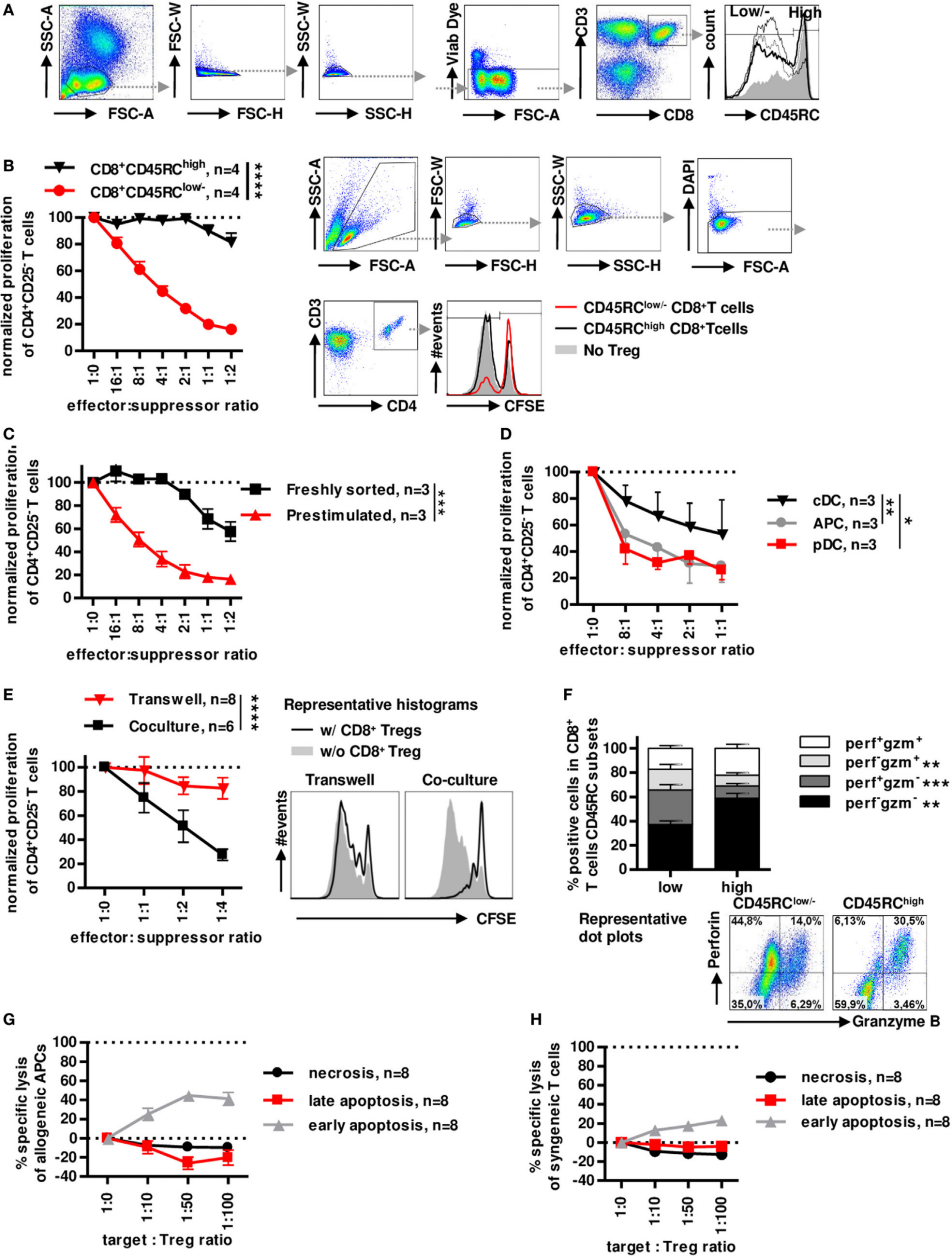
Low expression of CD45RC in CD8^+^ T cells positively correlates with suppressive activity, but not cytotoxicity. PBMCs from healthy volunteers were analyzed for the phenotype of CD8^+^ Tregs. **(A)** CD8^+^ Tregs were defined by gating on lymphocytes morphology, FSC and SSC singlets, living cells, CD3 and CD8 double positive cells, and CD45RC^low/−^ cells including negative and intermediate expression of CD45RC marker. Far right: histogram represents overlay of CD45RC expression by four healthy volunteers. **(B)** CD8^+^CD45RC^low/−^ T cells (red lines) and CD8^+^CD45RC^high^ T cells (black line) from fresh PBMCs of healthy volunteers were sorted and stimulated overnight with anti-CD3 and anti-CD28 MAbs and tested for suppressive activity on proliferation of syngeneic CD4^+^CD25^−^ T cells stimulated with allogeneic APCs, in a range of effector:suppressor ratio. Proliferation was normalized to proliferation in the absence of Tregs. Two-way row-matched (RM) ANOVA, *n* = 4 for each group, ****p < 0.0001. Representative histograms of T CD4^+^ responder cell proliferation in the presence of CD8^+^CD45RC^low/−^ (red line) and CD8^+^CD45RC^high^ (black line) T cells or without CD8^+^ T cells (filled gray), after gating on morphology, excluding doublet cells, and gating on living CD4^+^ T cells. **(C)** CD8^+^CD45RC^low/−^ T cells from fresh PBMCs of healthy volunteers were sorted and stimulated overnight with anti-CD3 and anti-CD28 MAbs (red lines) or not (black line) and tested for suppressive activity on proliferation of syngeneic CD4^+^CD25^−^ T cells stimulated with allogeneic APCs, in a range of effector:suppressor ratio. Proliferation was normalized to proliferation in the absence of Tregs. Two-way RM ANOVA, *n* = 3 for each group, ***p < 0.001. **(D)** Plasmacytoid dendritic cells (pDCs), conventional dendritic cells (cDCs), and total APCs were compared as stimulator cells for suppressive activity of Tregs. Proliferation was normalized to proliferation in the absence of Tregs. Two-way RM ANOVA, *n* = 3, *p < 0.05, **p < 0.01. **(E)** CD8^+^CD45RC^low/−^ Tregs were sorted, stimulated overnight with anti-CD3 and anti-CD28 MAbs, and compared for suppressive activity when physically separated from responder cells by a 0.4 µm transwell membrane (transwell, *n* = 8) vs. in contact with responder cells (coculture, *n* = 6). Proliferation was normalized to proliferation in the absence of Tregs with or without transwell membrane. APCs were added in both compartments. Two-way RM ANOVA, ****p < 0.0001. **(F)** CD45RC^low/−^ and ^high^ CD8^+^T cells were compared for granzyme and perforin expression after PMA-ionomycine stimulation. Wilcoxon matched-pairs signed rank test two-tailed, *n* = 15, **p < 0.01, ***p < 0.001. Bottom: Representative dot plots of perforin and granzyme expression in CD45RC^low/−^ (left) and CD45RC^high^ (right) CD8^+^ T cells. **(G)** CD8^+^CD45RC^low/−^ Tregs were tested for specific lysis of allogeneic APCs in a range of target:Tregs ratio. Necrosis (black line) and late (red line) and early (gray line) apoptosis were defined by annexin V and Dapi labeling after 15 h of coculture. *n* = 8. **(H)** CD8^+^CD45RC^low/−^ Tregs were tested for specific lysis of syngeneic CD4^+^CD25^−^ T cells in a range of target:Tregs ratio. Necrosis (black line) and late (red line) and early (gray line) apoptosis were defined by Annexin V and Dapi labeling after 15 h of coculture. *n* = 8.

Similarly to rats (8, 37, 38), the CD45RC marker is differently expressed on CD8^+^ T cells in healthy individuals (Figure 1A) with no relation to age or gender (Figure S1A,B in Supplementary Material) and can identify two subsets. We have previously shown that CD45RC expression is not redundant with CD45RA, CD45RB and CD45RO expression, particularly for CD8^+^ and CD4^+^ Tregs (25). Prestimulated sorted CD8^+^CD45RC^low/−^ Tregs showed a dose-dependent suppression of CD4^+^ T cell proliferation (>80% at a 1:1 effector:suppressor ratio), in contrast, prestimulated sorted CD8^+^CD45RC^high^ T cells did not significantly inhibit allogeneic CD4^+^ T cell proliferation (Figure 1B), establishing the suppressive capacity of CD8^+^CD45RC^low/−^ T cells. Freshly sorted unstimulated CD8^+^CD45RC^low/−^ Tregs were less suppressive (30% at a 1:1 effector:suppressor ratio) than CD8^+^CD45RC^low/−^ Tregs prestimulated with a short 12 h anti-CD3/28 stimulation (Figure 1C), suggesting that stimulation is important for CD8^+^ Tregs to exert efficient suppression. CD8^+^CD45RC^low/−^ Tregs isolated from frozen PBMCs also exhibited a suppressive activity (Figure S1C in Supplementary Material).

As in the rat we previously demonstrated the preferential interaction of CD8^+^CD45RC^low/−^ Tregs with pDCs for an optimal suppressive activity (39), we tested the suppressive potential of CD8^+^CD45RC^low/−^ Tregs in the presence of cDCs (CD3^−^CD19^−^CD1c^+^Nrp-1^−^) and pDCs (CD3^−^CD19^−^CD1c^−^Nrp-1^+^) sorted simultaneously, in comparison to total MHC class II^+^ APCs as stimulator cells (Figure 1D). CD8^+^CD45RC^low/−^ Tregs were significantly more suppressive in the presence of pDCs compared to cDCs and total APCs.

To determine a contact-dependent suppressive activity, we assessed the suppressive activity of CD8^+^CD45RC^low/−^ Tregs in transwell experiments with CFSE-labeled CD4^+^CD25^−^ T cells plus allogeneic APCs in the lower chamber and CD8^+^CD45RC^low/−^ Tregs plus the same allogeneic APCs in the upper chamber (Figure 1E). Surprisingly, CD8^+^CD45RC^low/−^ Tregs completely lost their suppressive activity when separated from effector T cells, demonstrating the requirement for cell contact. Finally, analysis of perforin (perf) and granzyme B (gzm) demonstrated a very different profiles between CD45RC^low/−^ versus to CD45RC^high^ T cells with significantly less perf^−^gzm^−^ and perf^+^gzm^+^ cells and more single positive perf^+^gzm^−^ or perf^−^gzm^+^ in CD8^+^CD45RC^low/−^ T cells compared to CD45RC^high^ T cells (Figure 1F). To further analyze cytolysis as a suppressive mechanism, we analyzed early apoptosis (Dapi^−^AnnexinV^+^), late apoptosis (Dapi^+^AnnexinV^+^), and necrosis (Dapi^+^AnnexinV^−^) of APCs or CD4^+^ T cells in the presence of increasing ratio of CD8^+^CD45RC^low/−^ Tregs for a long incubation of 15 h (Figure 1G, APCs and Figure 1H CD4^+^ T cells). We observed no necrosis or late apoptosis induction toward targets, but only a low early apoptosis induction toward both targets at very high target:effector ratios (Figures 1G,H) which is in contrast with the suppression observed at low target:effector ratios. The low apoptosis induction suggests that cytotoxic mechanisms play little role in the suppressive effect mediated by the CD8^+^CD45RC^low/−^ Tregs.

Altogether, these data demonstrate that CD8^+^CD45RC^low/−^ Tregs exhibits a highly suppressive activity in contrast to CD8^+^CD45RC^high^ T cell and that CD8^+^CD45RC^low/−^ Tregs act in a contact-dependent manner but do not use cytolysis to kill effector T cells or APCs.

### CD8^+^CD45RC^low/−^ Tregs Cells Express Foxp3 and Secrete IFNγ, IL-10, IL-34, and TGFβ to Inhibit Anti-Donor Immune Responses

**Figure 2:**
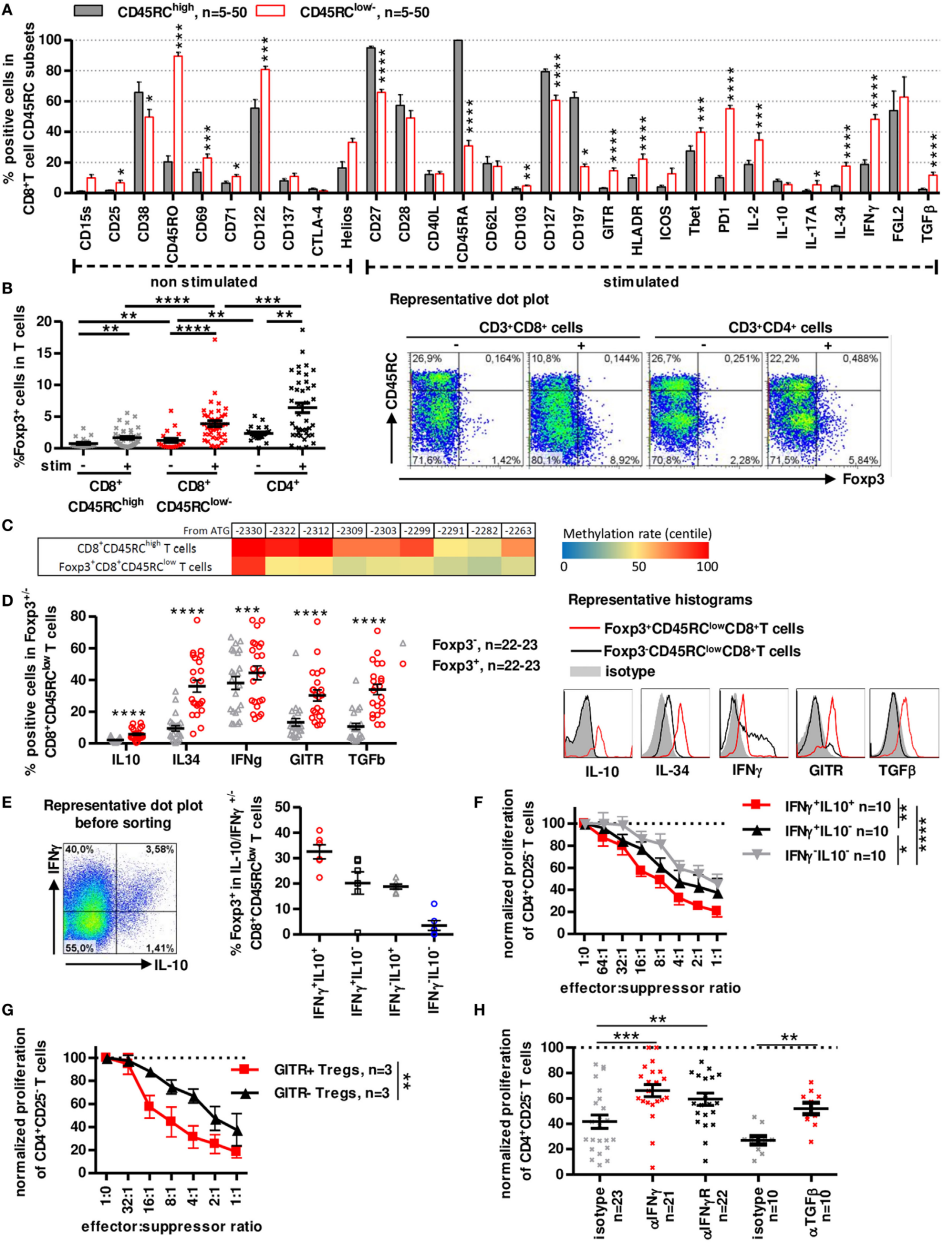
A distinct subset of human CD8^+^CD45RC^low/−^ T cells expresses Foxp3 and secretes IFNγ, IL-10, IL-34, and TGFβ to inhibit antidonor immune responses. **(A)** CD45RC^low/−^ and CD45RC^high^ subsets of blood CD8^+^ T cells were analyzed and compared for expression of activation markers, cytokines and Treg-associated markers after a 7 h PMA-ionomycin stimulation where indicated. Wilcoxon matched-pairs signed rank test two-tailed, *n* = 5–50. **(B)** Foxp3 expression was analyzed in unstimulated or PMA-ionomycin stimulated CD8^+^ CD45RC^low/−^ and CD45RC^high^ T cells and CD4^+^CD25^+^CD127^low/−^ Tregs. Wilcoxon matched-pairs signed rank test two-tailed, *n* = 39, **p < 0.01, ***p < 0.001, ****p < 0.0001. Right: Representative dot plot of Foxp3 and CD45RC staining on T cells. **(C)** FOXP3 TSDR methylation of stimulated CD8^+^CD45RC^low/^Foxp3^+^ and CD45RC^high^ T cells. Color coding represents centile percentage methylation. **(D)** Left. Markers differentially expressed in Foxp3^+^ vs. Foxp3^−^ CD8^+^CD45RC^low/−^ T cells. Wilcoxon matched-pairs signed rank test two-tailed, *n* = 22–23, ***p < 0.001, ****p < 0.0001. Right, Representative histograms of GITR and cytokines **(E)** Foxp3 expression was analyzed in sorted IFNγ/IL10 producing CD8^+^CD45RC^low/−^ T cells, purity was >98%. Graph represents mean ± SEM of Foxp3^+^ cells percentage in IL-10^+^IFNγ^+^, IL-10^-^IFNγ^+^, IL-10^+^IFNγ^-^, and IL-10^-^IFNγ^−^ CD8^+^CD45RC^low/−^ T cells. Left: Representative dot plot of IFNγ and IL-10 expression in CD8^+^CD45RC^low/−^ T cells. **(F)** CD8^+^CD45RC^low/−^ Tregs were sorted from healthy volunteers fresh blood, stimulated overnight with anti-CD3 and anti-CD28 MAbs, sorted again on IFNγ and IL-10 secretion and tested for suppressive activity in a range of effector:suppressor ratio. Proliferation was normalized to proliferation in the absence of Tregs. Two-way row-matched (RM) ANOVA, *n* = 10, *p < 0.05, **p < 0.01, ****p < 0.0001. **(G)** CD8^+^CD45RC^low/−^ Tregs were sorted from healthy volunteers’ fresh blood, stimulated overnight with anti-CD3 and anti-CD28 MAbs, sorted again on GITR expression and tested for suppressive activity in a range of effector:suppressor ratio. Proliferation was normalized to proliferation in the absence of Tregs. Two-way RM ANOVA, *n* = 3, **p < 0.01. **(H)** Blocking Abs to TGFβ, IFNγ, IFNγ-R were added at day 0 of coculture. Proliferation in the presence of freshly sorted CD8^+^CD45RC^low/−^ Tregs was normalized to proliferation in the absence of Tregs. Wilcoxon matched-pairs signed rank test, two-tailed, left, *n* = 21–23; right, *n* = 10, **p < 0.01, ***p < 0.001.

To determine the phenotype of CD8^+^CD45RC^low/−^ Tregs and compare its phenotype to previously described Treg subsets, we examined expression of various cell surface markers, intracellular cytokines, chemokine receptors, and transcription factors (Figure 2) and tested their role in suppressive assays.

Phenotypic analysis of freshly isolated CD8^+^CD45RC^low/−^ T cells using CD28, CD27, CD45RA, and CCR7 markers identifying naive and different memory subsets (40, 41) (Figure S2A in Supplementary Material), showed that CD8^+^CD45RC^low/−^ T cells contained mostly memory cells with predominant effector-memory type 1 subset (i.e., CCR7^-^CD45RA^-^CD27^+^CD28^+^) with 75% CD45RA^−^ and >50% CD27^+^CD28^+^ expression while CD45RC^high^ T cells were primarily naive and T effector-memory RA cells. Detailed phenotypic characterization using flow cytometry revealed in CD8^+^CD45RC^low/−^ T cells a significantly higher expression of CD25, CD45RO, CD69, CD71, CD103, CD122, GITR, HLA-DR, Tbet, PD-1, IL2, IL-17a, IL-34, IFNγ, and TGFβ1 and inferior expression of CD27, CD38, CD45RA, CD127, and CCR7 by CD8^+^CD45RC^low/−^ Tregs compared to CD8^+^CD45RC^high^ T cells (Figure 2A; Figure S2B in Supplementary Material). Markers that were present in >50% of freshly isolated CD8^+^CD45RC^low/−^ Tregs were CD27, CD45RO, CD122, CD127, PD-1, and FGL-2 (Figure 2A; Figure S2B in Supplementary Material), CD28^low^ and CD122^+^ CD8^+^ T cell subsets, previously described as CD8^+^ Tregs in rodents and human (42, 43) did not correlate with CD8^+^CD45RC^low/−^ T cells but were rather evenly distributed among CD8^+^CD45RC^low/−^ or CD45RC^high^ T cells (44–46).

Analysis of Foxp3 demonstrated that Foxp3^+^ cells were mainly in CD8^+^CD45RC^low/−^ Tregs compared to CD8^+^CD45RC^high^ T cells and this was particularly evident following a short stimulation with 1.26 ± 0.32 before stimulation to 3.87 ± 0.47 after stimulation for CD8^+^CD45RC^low/−^ (Figure 2B). Expression of Foxp3 was lower in CD8^+^CD45RC^low/−^ Tregs than in canonical CD4^+^CD25^+^CD127^−^ Tregs (2.35 ± 0.25 before stimulation to 6.41 ± 0.76 after stimulation for CD4^+^CD25^+^CD127^−^ Tregs). Foxp3 was evenly distributed in naive and memory subsets of CD8^+^ Tregs (Figure S2C in Supplementary Material). In depth analysis of DNA methylation pattern of the Foxp3 gene using targeted next-generation bisulfite sequencing revealed a demethylation at the different CpG sites in sorted CD8^+^CD45RC^low^Foxp3^+^ Tregs compared to CD8^+^CD45^high^ T cells, suggesting a stable expression of Foxp3 (Figure 2C). We further focused on Foxp3 marker and observed that Foxp3^+^CD45RC^low/-^CD8^+^ T cells compared to Foxp3^-^CD45RC^low/-^CD8^+^ T cells showed not only significantly superior expression for IL-10, IL-34, IFNγ, GITR, and TGFβ but also that with the exception of IFNγ all were almost not expressed by Foxp3^-^CD45RC^low/-^CD8^+^ T cells (Figure 2D). Of note, IFNγ MFI levels remained low in Foxp3^+^CD45RC^low/-^CD8^+^ T cells in contrast to Foxp3^-^CD45RC^low/-^CD8^+^ T cells (Figure 2D representative histogram). We have described that rat CD8^+^CD45RC^low/−^ T cells expresses IL-10 and mediate their suppressive function at least by the expression of IFNγ (8). Moreover, we observed that a fraction of IFNγ expressing cells among human CD8^+^CD45RC^low/−^ cells also express IL-10 and that cells coexpressing IFNγ and IL-10 were the most frequent among CD8^+^CD45RC^low/-^Foxp3^+^ T cells (Figure 2E). Sorting of live CD8^+^CD45RC^low/−^ Tregs based on IFNγ/IL-10 secretion resulted in identification of a subset with a superior suppressive capacity in IFNγ^+^IL-10^+^ CD8^+^CD45RC^low/−^ Tregs compared to their less efficient IFNγ^−^ or IL-10^−^ counterparts (Figure 2F). Given that GITR was also preferentially expressed by Foxp3^+^ cells, we sorted GITR^+^ and GITR^−^ CD8^+^CD45RC^low/−^ Tregs and observed that GITR^+^CD8^+^CD45RC^low/−^ Tregs had a significantly superior suppressive activity than their GITR^−^ counterparts (Figure 2G). Other surface markers such as CD38, HLA-DR, CD45RA, CD127, CD197, CD27, CD28, and CD25 that were used to separate a positive and negative fraction did not allow identification of a significantly more suppressive subset (Figure S3A in Supplementary Material). To address the role of the cytokines in the suppressive activity of the CD8^+^CD45RC^low/−^ Tregs, we added blocking anti-cytokine or anti-cytokine-receptor antibodies in the suppressive assay, compared to isotype controls (Figure 2H; Figure S3B in Supplementary Material). We previously demonstrated the involvement of IL-34 in human and rat CD8^+^CD45RC^low/−^ and of IFNγ in rat CD8^+^CD45RC^low/−^ Tregs suppressive activity (27). The present data demonstrate a role for both IFNγ and TGFβ (Figure 2H), but not IL-4, IL-10, and IL-13 (Figure S3B in Supplementary Material), in the suppressive activity of human CD8^+^CD45RC^low/−^ Tregs, since each blocking antibody partially restored effector T cells proliferation. Suppression by Tregs did not depend on IL-2 deprivation as addition of high amount of exogenous IL-2 only slightly restored effector T cell proliferation (Figure S3C in Supplementary Material). Inhibition of other suppressive molecules such as CTLA-4, ICOS, HO-1, IDO, or NOs in the suppressive assay did not restore proliferation of Teff cells (Figure S3D in Supplementary Material).

Altogether, these data suggest that CD8^+^CD45RC^low/−^ Tregs have a phenotype of memory-like cells and contain a subpopulation of activated cells expressing Foxp3^+^, IFNγ^low^, IL-10^+^, IL-34^+^, and TGFβ^+^, key molecules of Tregs (8, 27) and act through cytokines secretion.

### Expanded CD8^+^CD45RC^low/−^ Tregs Showed Increased Suppressive Function and Addition of Rapamycin during Expansion Improved This Effect

**Figure 3:**
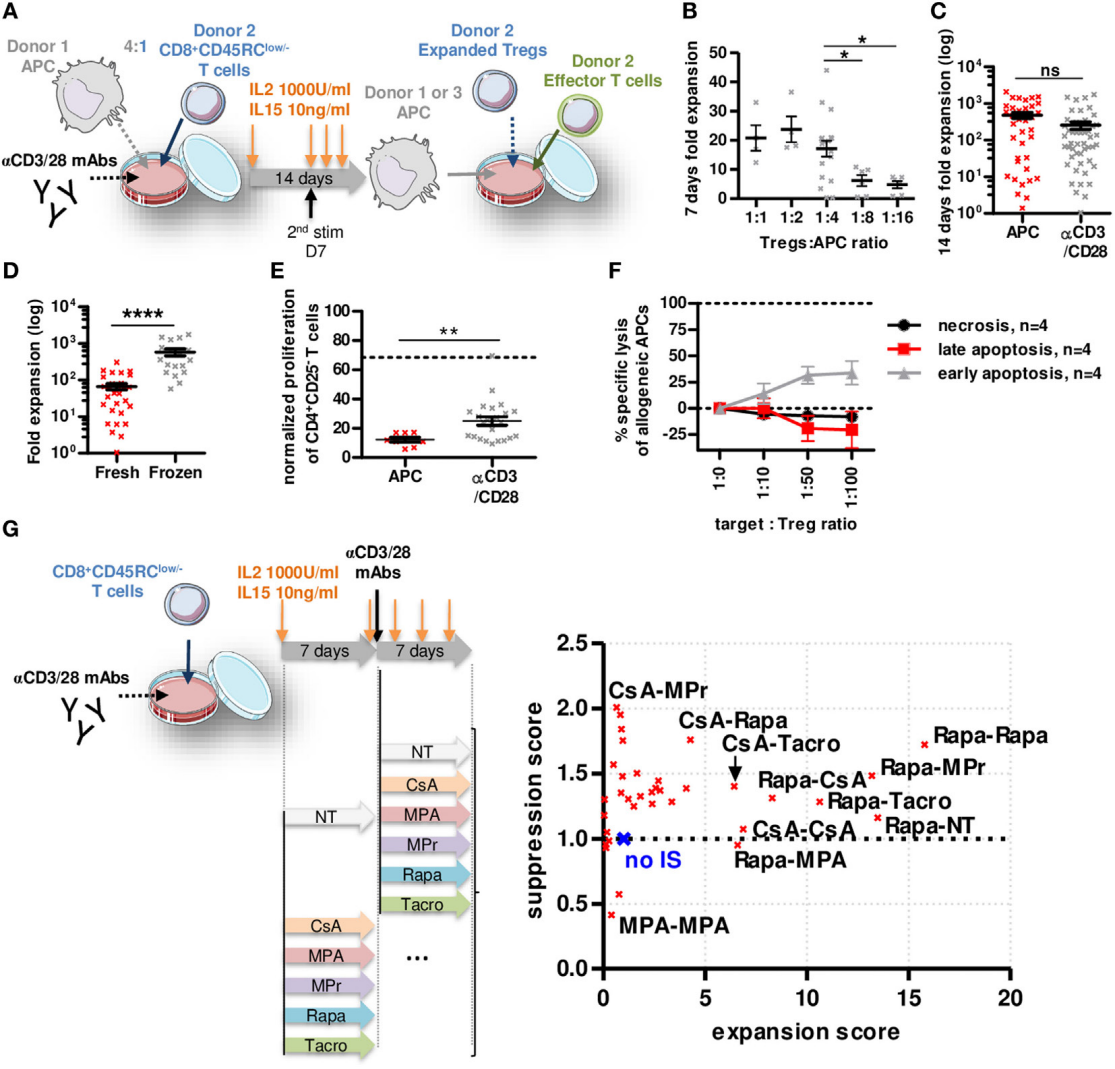
Rapamycin improved non cytotoxic CD8^+^CD45RC^low/−^ Tregs expansion and function. **(A)** Model depicting CD8^+^CD45RC^low/−^ Tregs expansion with allogeneic APCs or anti-CD3/CD28 MAbs at d0, restimulated at d7 with anti-CD3/CD28 MAbs and analyzed at d14. **(B)** Expansion yield of CD8^+^CD45RC^low/−^ Tregs was analyzed in a range of Tregs:allogeneic APCs ratio after 7 days culture. Mann–Whitney two-tailed test, *n* = 6–18, *p < 0.05. **(C)** CD8^+^CD45RC^low/−^ Tregs were stimulated at d0 with allogeneic APCs (*n* = 37) or anti-CD3/CD28 MAbs (*n* = 49). **(D)** CD8^+^CD45RC^low/−^ Tregs were sorted from fresh (*n* = 31) or thawed PBMCs (*n* = 18) and compared for expansion fold at d14. **(E)** CD8^+^CD45RC^low/−^ Tregs were expanded with allogeneic APCs or anti-CD3/CD28 MAbs for 7 days and tested for suppression on syngeneic CD4^+^ T cells proliferation at a 1:1 cell ratio compared to fresh CD8^+^CD45RC^low/−^ Tregs (dotted line). Mann–Whitney two-tailed test **p < 0.01. **(F)** 7 days APC-expanded CD8^+^CD45RC^low/−^ Tregs were tested for lysis toward same APCs in a range of target:Tregs ratio. **(G)** CD8^+^CD45RC^low/−^ Tregs were expanded for 14 days with anti-CD3/CD28 MAbs supplemented with immunosuppressors or not (NT) and assessed for suppressive activity and expansion yield. Each point represents mean of four independent experiments normalized to Tregs expanded without drug. (no IS, blue cross).

To determine whether CD8^+^CD45RC^low/−^ Tregs are suitable for a clinical trial, we set up an expansion protocol using sorted total CD8^+^CD45RC^low/−^ Tregs (Figure 3A; Figure S4A in Supplementary Material) in the presence of different ratios with allogeneic APCs (Figure 3B) compared to a polyclonal stimulation (anti-CD3/28) and high dose of IL-2 (1,000 U/ml) and IL-15 (10 ng/ml) (Figure 3C). Expansion at ratio 1:4 with APCs or anti-CD3/28 resulted in equivalent expansion yield, up to 2,000-fold in 14 days (Figures 3B,C). Interestingly, CD8^+^CD45RC^low/−^ Tregs isolated from frozen PBMCs expanded 10-fold more than CD8^+^CD45RC^low/−^ Tregs isolated from freshly isolated PBMCs (Figure 3D). Importantly, both allogeneic and anti-CD3/28 expanded CD8^+^CD45RC^low/−^ Tregs were significantly more efficient at suppressing an allogeneic immune response compared to fresh CD8^+^CD45RC^low/−^ Tregs (at a 1:1 cell ratio, 88 and 75 vs. 32% suppression, respectively) (Figures 1C and 3E). CD8^+^CD45RC^low/−^ Tregs sorted from fresh or thawed PBMCs were equally suppressive following expansion (data not shown). Expanded CD8^+^CD45RC^low/−^ Tregs did not increase their cytotoxic activity compared to fresh CD8^+^CD45RC^low/−^ Tregs toward allogeneic APCs (Figures 1G and 3F), demonstrating that the superior suppressive capacity acquired upon expansion was not due to increased killing activity of the CD8^+^CD45RC^low/−^ Tregs. We tested the effect of immunosuppressive drugs (IS) (at concentrations used in the clinic) on Tregs survival 7 days following the 14 days expansion (Figure S4B in Supplementary Material) and observed no significant effect on Tregs survival. Next, we tested the effect of the ISs during the 14 days expansion on the expansion yield and suppressive function score of CD8^+^CD45RC^low/−^ Tregs (Figure 3G; Figure S4C in Supplementary Material). Each IS was added or not (NT) during 14 days or combined together subsequently (i.e., days 0–7 CsA, then days 7–14 MPr is labeled CsA-MPr in the upper left corner of Figure 3G). We observed a striking beneficial effect of Rapa on both expansion fold and suppression capacity when the expansion was performed during 14 days in the presence of Rapa or if Rapa was at least used during the first 7 days of the expansion (Rapa-MPr, Rapa-Tacro, Rapa-NT, Rapa-CsA) (Figure 3G, right; Figure S4C in Supplementary Material). In contrast, the presence of MPA inhibited the expansion of the CD8^+^CD45RC^low/−^ Tregs and decreased suppression (Figure 3G, right; Figure S4C in Supplementary Material).

Altogether, our data demonstrate here that CD8^+^CD45RC^low/−^ Tregs can be efficiently expanded, and that Rapa might be beneficial to improve expansion and function of CD8^+^CD45RC^low/−^ Tregs *in vitro*.

### Characterization of CD8^+^CD45RC^low/−^ Tregs following Expansion Compared to Fresh Ones Shows a Selective Regulatory Genes Expression

**Figure 4:**
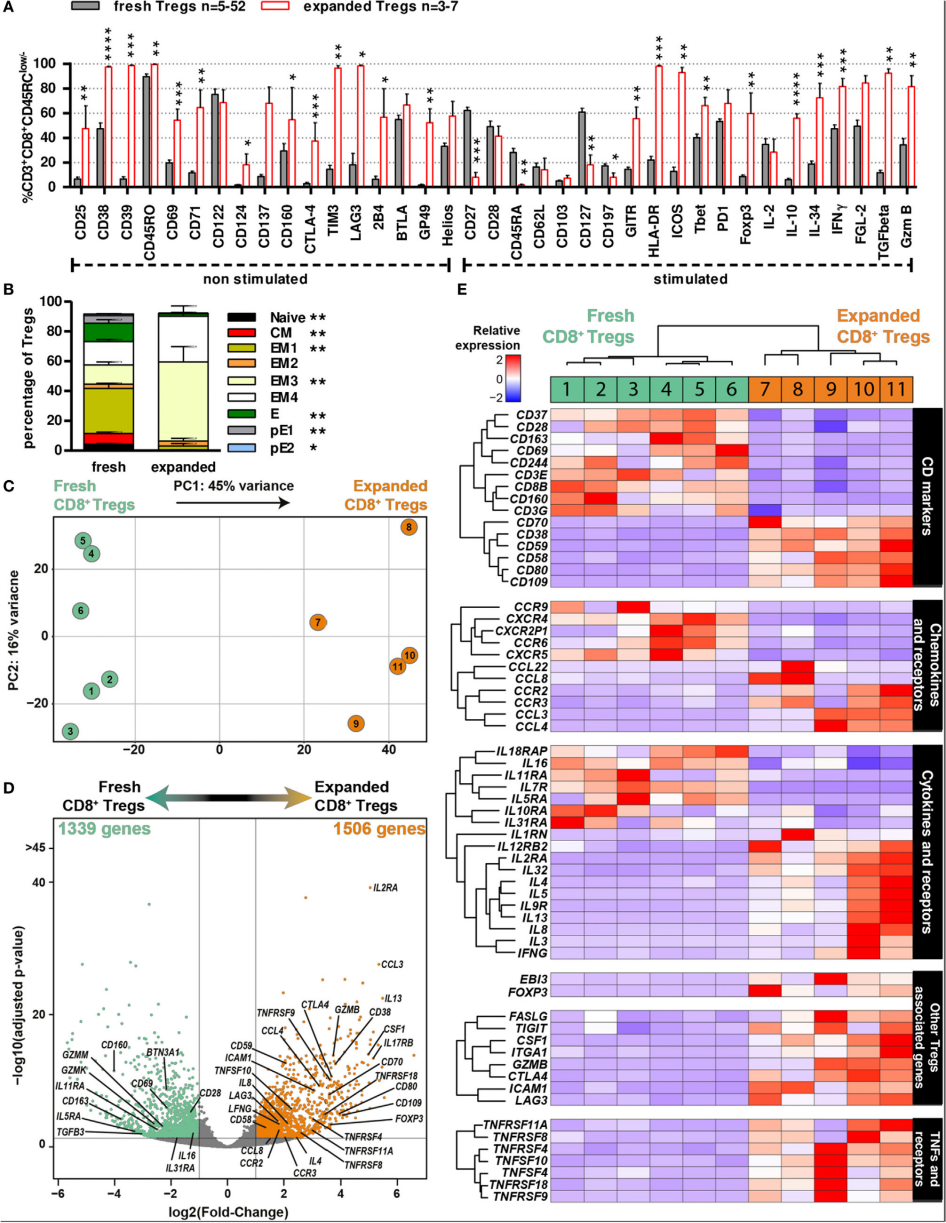
Phenotypic and transcriptomic profiling of CD8^+^CD45RC^low/−^ Tregs before and after expansion highlight a distinct signature for/of the cell therapy product. **(A)** CD8^+^CD45RC^low/−^ Tregs were expanded with anti-CD3/CD28, IL-2, and IL-15 for 14 days and analyzed for expression of activation and exhaustion markers or cytokines and Treg-associated markers after a 7 h PMA-ionomycin stimulation when indicated (*n* = 3–7) and compared to non-expanded CD8^+^CD45RC^low/−^ Tregs (*n* = 5–52). Mann–Whitney two-tailed test, *p < 0.05, **p < 0.01, ***p < 0.001, ****p < 0.0001. **(B)** CD8^+^CD45RC^low/−^ Tregs were analyzed for differentiation state basing on CCR7, CD45RA, CD27, and CD28 expression after expansion (“expanded,” *n* = 5) as compared to before expansion (“fresh,” *n* = 14), both without stimulation. Mann–Whitney two-tailed test, *p < 0.05, **p < 0.01. **(C)** Principal component analysis (PCA) of fresh vs. expanded CD8^+^CD45RC^low/−^ Treg. **(D)** Volcano plot representation of differential expression between fresh and expanded Tregs. Genes were colored when considered as differentially expressed, with adjusted *p*-value < 0.05 and two fold change. Using fresh CD8^+^CD45RC^low/−^ Tregs as a reference, green genes are downregulated and orange genes upregulated. **(E)** 3′ digital gene expression RNA-sequencing analysis was performed on CD8^+^CD45RC^low/−^ Tregs before and after expansion for 14 days. Expression levels of differentially expressed genes are presented as a heatmap; low expression levels are in blue, mean expression levels are in white and high expression levels are in red. Individual samples are numbered 1–11.

To better define expanded CD8^+^CD45RC^low/−^ Tregs that are to be used as cell therapy in transplantation, we performed flow cytometry analysis of the previously examined markers and 3′ digital expression RNA-Seq (DGE-Seq). Phenotypic analysis revealed that expanded CD8^+^CD45RC^low/−^ Tregs were highly enriched for expression of molecules involved in the function of CD4^+^ Tregs and of CD8^+^ Tregs before expansion (Figure 2), such as Foxp3, CD25, CD38, CD39, CTLA-4, GITR, TIM3, LAG3, ICOS, Foxp3, IL-10, IL-34, IFNγ, and TGFβ (Figure 4A; Figure S5A in Supplementary Material) but others like CD28, PD-1, or CD122 were unchanged and CD127 was decreased. In addition, expanded CD8^+^CD45RC^low/−^ Tregs displayed a more differentiated effector memory cell phenotype as defined by CD27^-^CCR7^-^CD45RA^−^ CD8^+^ Tregs vs. freshly isolated ones (Figure 4B). Altogether, the phenotypic profile suggests that the expansion process enriched in CD8^+^CD45RC^low/−^ Tregs with a regulatory signature. Hierarchical clustering, principal component and Pearson correlation analyzes highlighted the transcriptional changes following expansion (Figures 4C,D; Figure S5B in Supplementary Material). Indeed, DGE-Seq transcriptional profiling demonstrated that 1,506 genes were upregulated and 1,339 genes were downregulated in expanded versus freshly purified CD8^+^CD45RC^low/−^ Tregs (Figure 4D). When assessing the transcriptional changes, we considered that changes reflected an enhanced function and were involved in CD8^+^CD45RC^low/−^ Tregs activity. An important change in the transcriptional regulators involved in CD8^+^CD45RC^low/−^ Tregs mediated activity was the up-regulation of *FOXP3*, suggesting that CD8^+^CD45RC^low/−^ Tregs depend on Foxp3, as CD4^+^ Tregs do (Figure 4E). As *FOXP3*, genes commonly associated with Tregs suppressive function such as *CTLA4, GITR, LAG3, IFNG, GZMB, IL2RA* (*CD25*), *CD38, TNFRSF9* (*CD137*) were upregulated both at transcriptomic and proteomic levels, while *IL7RA* (*CD127*) was downregulated. Other genes were related to surface markers and costimulation molecules such as *TNFRSF9 (4-1BB), TNFRSF4 (OX40L), TNFRSF11A (RANK), TNFRSF8 (CD30), CD70, CD38, CD59, CD109, CD80*, and *CD58* were upregulated, while genes associated with activation such as *CD28* and *CD69* were significantly downregulated (Figure 4E). To gain insight into the mechanisms of action of expanded CD8^+^CD45RC^low/−^ Tregs, we analyzed in detail the cytokines, chemokines, and granzymes that are overexpressed. We found a panel of cytokines including IFNγ and others such as *CSF1* that we have observed upregulated by CD8^+^CD45RC^low/−^ Tregs in a model of tolerance in rat transplantation (25), as well as a number of chemokines associated with Tregs and ligand to *CCR5* including *CCL3, CCL4, CCL8*, as well as the chemokine receptors *CCR3* and *CCR2* influencing the migration pattern of Tregs. In contrast, although we observed the upregulation of *GZMB* (granzyme), we observed the downregulation of *GZMK* and *GZMM* and unaltered expression of *PRF-1* (perforin).

Altogether, our data demonstrate a distinct phenotype and transcriptional signature of CD8^+^CD45RC^low/−^ Tregs following expansion, supporting a potential increased suppressive activity with several regulatory genes and consolidate the demonstration that the expansion process adequately enriched in CD8^+^CD45RC^low/−^ Tregs with an improved regulatory activity suitable for transplantation.

### Polyclonal Expanded CD8^+^CD45RC^low/−^ Tregs Efficiently Delay Xenogeneic GVHD and Allogeneic Human Skin Graft Rejection in Immune Humanized Mice

**Figure 5:**
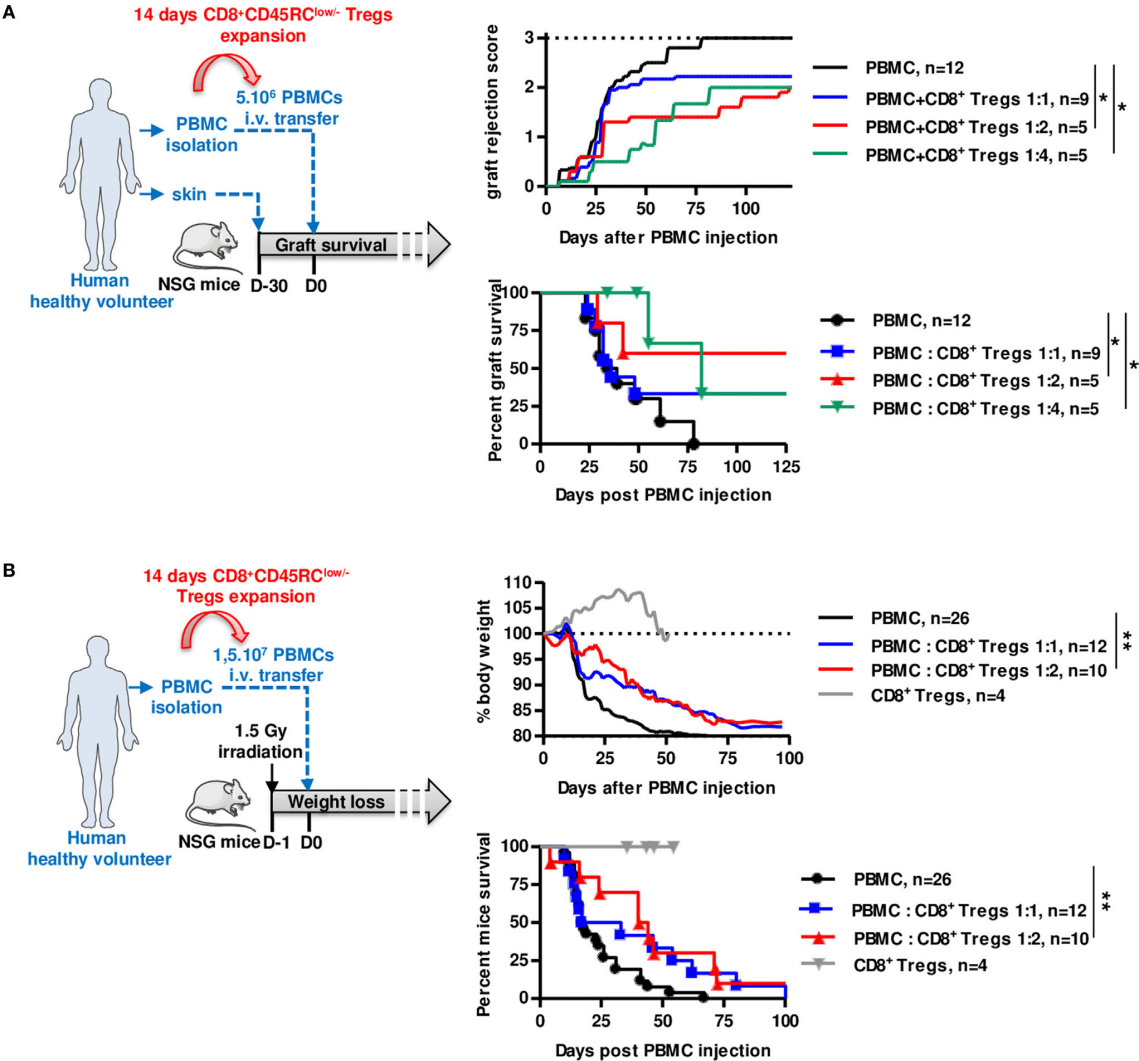
Expanded CD8^+^CD45RC^low/−^ Tregs efficiently delay transplant rejection and GVHD in NSG mice. CD8^+^CD45RC^low/−^ Tregs were expanded with anti-CD3/CD28, IL-2, and IL-15 and tested in models of allograft rejection **(A)** and GVHD **(B)** in NSG mice. **(A)** Left: scheme depicting skin allorejection model. Top: graft survival was scored on macroscopic signs of graft rejection from 0 to 3 (rejection). Two-way row-matched (RM) ANOVA. Bottom: survival of the skin graft. Log Rank (Mantel Cox). **(B)** Left: scheme depicting GVHD model. Top: body weight loss was scored. Two-way RM ANOVA. Bottom: survival of mice. Log Rank (Mantel Cox). *p < 0.05, **p < 0.01.

We finally assessed the suppressive potential of expanded CD8^+^CD45RC^low/−^ Tregs using two distinct models of human immune responses in NSG (NOD-Scid-IL-2Rγ^-/-^) mice, rejection of human skin grafts following injection of allogeneic human PBMCs (Figure 5A; Figure S6A in Supplementary Material) and xenogeneic GVHD following injection of human PBMCs (Figure 5B). Interestingly, we observed in both cases that cotransfer of 14 days polyclonally expanded CD8^+^CD45RC^low/−^ Tregs significantly inhibited skin graft rejection (Figure 5A), as well as GVHD (Figure 5B) and for both in a dose-dependent manner at 1:2 and 1:4 ratios, demonstrating the potential of the CD8^+^CD45RC^low/−^ Tregs as a cell therapy. Importantly, expanded CD8^+^CD45RC^low/−^ Tregs administration alone did not trigger GVHD proving that they display low cytotoxic activity (Figure 5B). Mice were in all cases, including those that did not develop graft rejection, well engrafted with human leukocytes (Figure S6C in Supplementary Material).

**Figure 6:**
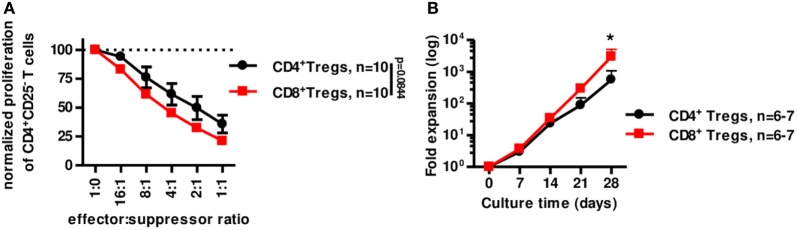
CD8^+^CD45RC^low/−^ Tregs are equivalent to canonical CD4^+^CD25^+^CD127^low/−^ Tregs for suppression *in vitro* but expand more efficiently. **(A)** CD8^+^CD45RC^low/−^ Tregs (*n* = 10) were compared to classical CD4^+^CD25^hi^CD127^low/−^ Tregs (*n* = 10) for suppressive activity in a range of effector:suppressor ratio after overnight anti-CD3 and anti-CD28 MAbs stimulation. Proliferation was normalized to proliferation in the absence of Tregs. Two-way row-matched (RM) ANOVA. **(B)** CD8^+^CD45RC^low/−^ Tregs (red line) and CD4^+^CD25^hi^CD127^low/−^ Tregs (black line) were sorted from fresh blood of healthy volunteers and compared for expansion yield when stimulated with anti-CD3 and anti-CD28 MAbs, IL-2, and IL-15 from d0 and every 7 days for 28 days. Two-way RM ANOVA test and Bonferroni posttest, *p < 0.05.

We next tested the suppressive capacity of CD8^+^CD45RC^low/−^ Treg *in vitro* compared to canonical CD4^+^CD25^high^CD127^low/−^ Tregs (Figure 6A). In similar conditions (i.e., fresh stimulated o/n Tregs) CD8^+^CD45RC^low/−^ Tregs were at least as suppressive as classical CD4^+^CD25^high^CD127^low/−^ Tregs (Figure 6A). In addition, CD8^+^CD45RC^low/−^ Tregs expanded significantly more than CD4^+^CD25^high^CD127^low/−^ Tregs in the presence of a polyclonal stimulation (Figure 6B). Interestingly, CD4^+^CD25^high^CD127^low/−^ Tregs from fresh or frozen PBMCs expanded similarly [while frozen CD8^+^CD45RC^low/−^ Tregs expanded more than fresh (Figure 3D)] and 10-fold less than CD8^+^CD45RC^low/−^ Tregs from frozen PBMCs (Figure S6B in Supplementary Material; Figure 3D).

Altogether, our data demonstrate here that CD8^+^CD45RC^low/−^ Tregs expand more efficiently in polyclonal conditions than CD4^+^CD25^high^CD127^low/−^ Tregs and are at least as efficient to suppress anti-donor immune responses *in vitro*.

## Discussion

We examined human CD8^+^ Tregs phenotype and function and potential in transplantation. Our data demonstrated that CD8^+^CD45RC^low/−^ Tregs used different suppressive mechanisms and were at least as efficient as CD4^+^CD25^high^CD127^low/−^ Tregs to inhibit anti-donor immune response *in vitro*. Identification of a master gene for the CD8^+^ Treg lineage is a long standing quest and Foxp3 or Helios have been controversial markers of CD8^+^ Tregs, despite some evidences in mice (10, 47). In rat, in a model of cardiac allotransplantation, we have observed that fresh CD8^+^CD45RC^low/−^ Tregs expressed ~2% of Foxp3 (8) and that Foxp3 was not upregulated following activation (28), however, the absence of Foxp3-deficient rat models remains a limitation for characterization of the role of Foxp3 in this model of transplantation. In mice, Cantor’s group has shown that Helios-deficient Qa-1-restricted CD122^+^CD8^+^ Tregs develop an unstable phenotype during inflammatory responses, suggesting an important role for the transcription factor Helios (47–49). Studies have demonstrated the existence of a Foxp3^+^ CD8^+^ Tregs in mice and human treated with low-dose IL-2 (16). Our data demonstrate that Foxp3, but also Helios, are coexpressed and contained within CD45RC^low/−^ IFNγ^low^ subset of CD8^+^ T cells and could constitute human lineage markers for CD8^+^ Tregs but further research is needed. Another limiting factor for CD8^+^ Tregs characterization is the lack of surface markers, in contrast to CD4^+^ Tregs for which CD25^high^ and CD127^low/−^ were recognized surface markers (50, 51) and which do not seem to correlate with human suppressor CD8^+^ Tregs. More recently CD15s, a sialyl Lewis x, was demonstrated identifying a most suppressive Foxp3^high^ subset of CD4^+^ Tregs but this marker was not differentially expressed between CD8^+^CD45RC^low/−^ or CD8^+^CD45RC^high^ (52). ICOS and HLA-DR are markers that have also been associated with higher regulatory function of CD4^+^ Tregs and used to discriminate subsets of regulatory CD4^+^ cells (53, 54). We observed here that these two markers are expressed at low levels before expansion but are significantly upregulated upon expansion, thus ICOS and HLA-DR cannot be used to discriminate subsets of CD8^+^ Tregs, but can be associated with higher suppressive function. Looking for CD8^+^ Tregs markers, we demonstrate that they are enriched within the CD45RC^low/−^ subset. We have recently demonstrated that CD45RC is not expressed, or at low level by Tregs (both CD4^+^ and CD8^+^ in rats and humans) and we have demonstrated that a short-term anti-CD45RC mAb therapy can induce transplant tolerance by depleting Teff while allowing activation and amplification of CD8^+^ and CD4^+^ Tregs (25). Our DGE-RNAseq data and flow cytometry analysis have evidenced a number of potential markers for Tregs including CTLA4, GITR, LAG3, and NRP1. GITR is a known marker for CD4^+^ Tregs that has been involved in thymic Treg (tTreg) differentiation and expansion in mice and humans (55). GITR has never been associated with CD8^+^ Tregs in humans. In mice, a population of IL-10-producing CD8^+^ Tregs controlling CD8^+^ Teff responses during influenza infection expressed higher level of GITR (56). Our data showed the specific coexpression of GITR, IL-10, IL-34, TGFβ, and IFNγ^low^ with Foxp3. This phenotypic profile suggests that part of CD8^+^ Tregs are natural thymic tTreg derived cells that are distinct from peripherally induced Tregs cells [including Helios (57)]. The absence of expression of the chemokine receptor CCR7 suggests a non-lymphoid tissue pattern of migration and an IL-2-independent survival mode (58), suggesting dependence on stimulatory receptor such as GITR and eventually migration into tissues controlled by other chemokines and receptors, such as CCL1 and CCR8 for CD4^+^ Tregs (59). High expression of CD38 or low expression of CD28 have been described previously in mice as markers of CD8^+^ Tregs (9, 60, 61), and accordingly we observed in DGE-RNASeq data that CD38 is upregulated and CD28 downregulated following expansion and enrichment in CD8^+^ Tregs. Memory like CD8^+^CD38^+^ T cells with regulatory properties have been shown to act through IFNg secretion and cell contact (60). CD8^+^CD45RC^low/−^ Tregs have a more complex profile of cytokine expression with secretion of IFNγ^low^ and IL-34, that we have demonstrated as critical in CD8^+^ Tregs function in rat (12, 27, 60, 62). In addition, we show that Foxp3^+^CD8^+^CD45RC^low/−^ Tregs all secrete but at low levels IFNγ (low MFI) in contrast to Foxp3^−^ Teff and that there is a subset of Tregs that dually secrete IFNγ and IL-10. In total, Foxp3^+^CD8^+^CD45RC^low/−^ Tregs express upon short stimulation IFNγ, IL-10 and IL-34 (about 5% of CD8 T cells) and are superior in number and slightly in function than CD4^+^CD25^high^CD127^low/−^ Tregs, which suggests an important role for this cell population and a high dependence on cytokine production for suppression. Nevertheless, other populations of Tregs may be contained within CD8^+^CD45RC^low/−^ since IFNγ^−^ and IL-10^−^ counterparts also displayed a suppressive activity. This finding raises the question of a preferential contact with pDCs similar to what we have demonstrated in the rat (39) and the role of antigen-specificity on Treg activation, expansion and suppressive activity. In contrast with our data showing cytokine mediated suppression, CD8^+^ Tregs required a cell contact with APCs, and preferentially pDCs at least, and a close proximity with Teff to inhibit antigen-mediated Teff activation as shown by transwell experiments. Although here we did not identify the exact cell-contact mechanism involved, previously we showed in the rat model of transplantation that suppressive activity of CD8^+^ Tregs in contact with CD4^+^ T cells was Fgl-2 and IFN-γ dependent (39). Collison et al. showed that a contact between Tconv and CD4 + Tregs was required for induction of suppression by Tregs in an IL-35- and IL-10-dependent manner (63). These features for antigen recognition for CD8^+^ Tregs were key to exert inhibitory activity and demonstrating that pDCs have superior protolerogenic activity (64, 65). The identification of antigens recognized by Tregs is an important objective to increase their activity and in a rat allograft transplantation model, we identified MHC class II donor-derived antigens presented by recipient MHC-I molecules to CD8^+^CD45RC^low/−^ Tregs (28). We also demonstrated that administration of these donor peptides induced CD8^+^ Tregs and transplant tolerance (28). The recent use of chimeric antigen receptor with donor HLA specificity has been recently used to redirect CD4^+^ Tregs bypassing the natural TCR (66–68).

We set up a protocol of expansion of CD8^+^CD45RC^low/−^ Tregs in the presence of high-dose IL-2 and IL-15. IL-15 is known growth factor of CD8^+^ T cells that promote survival and activation and a potent inducer of Tregs (both CD4^+^ and CD8^+^ Tregs) (24, 69–71). Another interesting finding was that Rapa might be beneficial to improve both expansion and function of CD8^+^CD45RC^low/−^ Tregs *in vitro*. Rapa has been shown to be beneficial *in vivo* for both CD8^+^ and CD4^+^ Tregs in mice by increasing stability of Foxp3 (72) and to increase numbers of CD8^+^CD28^−^ Tregs in kidney transplanted patients treated with Rapa (73). In the context of cell therapy in transplantation, our data suggest that expansion in the presence of Rapa and coadministration with Rapa could be beneficial. We demonstrated the potential of expanded CD8^+^CD45RC^low/−^ Tregs as a cellular therapy in two models of human immune responses in NSG humanized mice rejecting either allogeneic human skin graft or developing an acute xenogeneic GVHD. CD8^+^CD45RC^low/−^ Tregs could, in a dose-dependent manner, inhibit GVHD and skin graft rejection indefinitely for some of the recipients, suggesting even inhibition of chronic graft rejection and a high potential for CD8^+^ Tregs cell-based therapy. Alloantigen-specific CD4^+^ Tregs administered to NSG mice infused with xenogeneic PBMCs have been shown to efficiently delay clinical signs of GVHD and skin transplantation, while natural CD4^+^ Tregs or polyclonally expanded are much less efficient (74, 75) and antigen-specific CD8^+^ Tregs generated by either expansion with donor antigens or generation of CAR-CD8^+^ Tregs represent a promising future.

Thus, our findings have highlighted the potential for CD8^+^ Tregs in controlling immune responses in transplantation. Our results are supporting the use of CD8^+^ Treg cell therapy to treat transplant rejection.

## Ethics Statement

The 8–12-week-old NOD/SCID/IL2Rγ^-/-^ (NSG) mice were bred in our own animal facilities in SPF conditions (accreditation number C44-278) and this study was carried out according to permit number APAFIS 3168.

## Author Contributions

CG wrote the article, designed the research, and analyzed data. SB designed the research, performed research, and analyzed data. IA designed the research and analyzed data. LB, EA, EC, and LD performed the research and analyzed data. SK, AD, and VN-D performed the research. DM and JZ analyzed data. FB-W and FD contributed vital reagents.

## Conflict of Interest Statement

The authors declare that this study received funding from FINOX Biotech. The funder was not involved in the study design or collection, analysis, or interpretation of the data.
