# Supplementary material for: Ex Vivo Expanded Human Non-Cytotoxic CD8+CD45RClow/− Tregs Efficiently Delay Skin Graft Rejection and GVHD in Humanized Mice
Source: Front Immunol. 2018 Jan 31;8:2014. doi: 10.3389/fimmu.2017.02014 (PMC5797797; doi:10.3389/fimmu.2017.02014)
Supplement: Supplementary file 1 [file Data_Sheet_1.DOCX]

**Supplementary figure 1. CD45RC marker expression does not correlate with age and gender but negatively correlates with regulatory function even after thawing. (a,b)** Proportion of CD45RC^low/-^ cells in CD8^+^ T cells in blood of healthy volunteers was analyzed for correlation with age (**a**, Linear regression, n=33, ns) and gender (**b**, Mann Withney test, n=24 males and 8 females, ns) of healthy volunteers. **(c)** CD8^+^CD45RC^low/-^ T cells were sorted from fresh PBMCs or from thawed PBMCs, stimulated ON with anti-CD3 and anti-CD28 MAbs, and tested for suppressive activity in a range of effector: suppressor ratio. Proliferation was normalized to proliferation in absence of Tregs.

**Supplementary figure 2. Freshly isolated CD8^+^CD45RC^low/-^ Tregs phenotype.**

**(a)** Unstimulated CD45RC^low/-^ and ^high^ subsets in blood CD8^+^T cells were compared for differentiation status based on CCR7, CD45RA, CD27 and CD28 expression: CCR7 and CD45RA cells to define naive cells (N), central memory cells (CM), effector memory cells (EM), and terminally differentiated cells (TEMRA) then CD27 and CD28 to subdivide EM cells in type 1, 2, 3 and 4 EM cells, and TEMRA cells in pE1, pE2 and effector cells (E). Wilcoxon matched-pairs signed rank test two-tailed, n=10, *p<0.05, **p<0.01, ***p<0.001. **Right**: Representative dot plots of 10 healthy volunteers. **(b)** Representative histograms of expression of markers on freshly isolated PMA/ionomycine stimulated or not CD8^+^CD45RC^low/-^ T cells (red line), CD8^+^CD45RC^high^ T cells (black line), compared to isotypic control (filled grey**). (c)** Stimulated Foxp3^+^CD8^+^CD45RC^low/-^ Tregs in blood were compared for differentiation status based on CCR7, CD45RA, CD27 and CD28 expression: CCR7 and CD45RA cells to define naive cells (N), central memory cells (CM), effector memory cells (EM), and terminally differentiated cells (TEMRA).

**Supplementary figure 3. Freshly isolated CD8^+^CD45RC^low/-^ Tregs function.**

**(a)** CD8^+^CD45RC^low/-^ Tregs were sorted on marker expression from healthy volunteer fresh blood, stimulated ON with anti-CD3 and anti-CD28 MAbs, and tested for suppressive activity in a range of effector: suppressor ratio. Proliferation was normalized to proliferation in absence of Tregs. (**b)** Abs blocking IL-13 (n=4), IL-4 (n=4), IL-10 (n=18) or IL-10R (n=18) were added at d0 of co-culture. Proliferation in presence of freshly sorted CD8^+^CD45RC^low/-^ Tregs was normalized to proliferation in absence of Tregs. Mann Whitney, ns vs isotype mAbs. **(c)** IL-2 deprivation was assessed by complementing co-culture medium with 1000U/ml IL-2 and assessing proliferation of responder cells in a range of effector:suppressor cell ratio. Proliferation was normalized to proliferation in absence of Tregs. Wilcoxon matched-pairs signed rank test, ns. **(d)** Inhibitors of IDO (1-MT and MTHT), NOs (DNMMA), HO-1 (SnPP) or control buffers, or Abs blocking CTLA-4 or ICOS or isotype mAbs control were added at d0 of co-culture. Proliferation in presence of CD8^+^CD45RC^low/-^ Tregs was normalized to proliferation in absence of Tregs.

 **Supplementary figure 4. CD8^+^CD45RC^low/-^ Tregs sorting and survival in presence of IS. (a)** Representative gating strategy for CD8^+^CD45RC^low/-^ Tregs sorting. CD8^+^CD45RC^low/-^ Tregs were sorted by gating on cell morphology, SSC and FSC singlet cells, Dapi^-^ living cells, CD3^+^CD4^-^ cells, and low expression of CD45RC marker. Purity of sorted Tregs was greater than 97%. **(b)** CD8^+^CD45RC^low/-^ Tregs were sorted from fresh blood of HVs, expanded for 14d with anti-CD3 and anti-CD28 MAbs, then cultured for 7d in presence of allogeneic APCs in medium supplemented or not with IL-2 and IL-15 and immunosuppressive drugs and assessed for survival on the last 7 days. Values >1 means proliferation of cells. n=7. **(c)** CD8^+^CD45RC^low^ Tregs were sorted from PBMCs of healthy volunteers, stimulated at day 0 and day 7 with anti-CD3 and anti CD28 MAbs, cultured with different combinations of immunosuppressive drugs over 14 days culture with a change of culture medium at day 7, and analyzed for expansion yield **(left)** and suppressive activity on CD4^+^CD25^-^T cells stimulated with allogeneic APCs **(right).** Results are expressed as expansion fold and suppression score normalized to NT condition. Wilcoxon signed rank test vs. 1, n=8 for each group. *p<0.05.

**Supplementary figure 5. Expanded CD8^+^CD45RC^low/-^ Tregs phenotype and transcriptome.** CD8^+^CD45RC^low/-^ T cells were expanded with anti-CD3/28 mAbs, IL-2 and IL-15 during 14d and analyzed for their phenotype (a) and transcriptome (b-c). **(a)** Representative histograms of expression of markers on expanded CD8^+^CD45RC^low/-^ T cells (red line), fresh CD8^+^CD45RC^low/-^ T cells (black line), compared to isotypic control (filled grey). **(b)** Pearson’s correlation heatmap of fresh and expanded CD8^+^CD45RC^low/-^ Tregs. The dendrogram was calculated by unsupervised clustering. **(c)** 3’ DGE RNA-Seq analysis was performed on CD8^+^CD45RC^low/-^ Tregs before and after expansion with anti-CD3/CD28 mAbs, IL-2 and IL-15 for 14d. Expression levels of differentially expressed genes are presented as a heatmap; low expression levels are in blue, mean expression levels are in white and high expression levels are in red.

**Supplementary figure 6. Expanded CD8^+^CD45RC^low/-^ Tregs inhibit skin rejection in engrafted NSG mice. (a)** Macroscopic analysis of human skin graft > 150d after human PBMCs transfer with (Top) or without (Bottom) Tregs. **(b)** CD4^+^CD25^+^CD127^low/-^ Tregs were sorted from fresh (n=7) or thawed PBMCs (n=5) from healthy volunteers, stimulated with anti-CD3 and anti-CD28 MAbs at d0 and d7 of culture, and compared for expansion fold at d14. Mann Whitney test two tailed, ***p<0.001. **(c)** NSG mice were irradiated, injected with human PBMCs, and analyzed for human CD45^+^ cell populations engraftment in mice blood. Results are expressed in percentage of human cells in total PBMCs in mice. **Right**: Representative dot plots of mouse blood cells.
